# Supplementary material for: The genome sequence of the ichneunomid wasp, Ischnus inquisitorius (Muller, 1776) (Hymenoptera: Ichneumonidae)
Source: Wellcome Open Res. 2025 Aug 15;10:448. [Version 1] doi: 10.12688/wellcomeopenres.24737.1 (PMC12759271; doi:10.12688/wellcomeopenres.24737.1)
Supplement: Peer review history

# Reviewer report: Reviewer response for version 1

**DOI:** 10.21956/wellcomeopenres.27254.r139039

**Published:** 2026-01-02

**Authors:** Julien Varaldi, Sara Oukkal

**Affiliations:** 1 Universite Claude Bernard Lyon, Villeurbanne, Auvergne-Rhône-Alpes, France; 2 UMR CNRS 5558 - LBBE, Universite Claude Bernard Lyon 1, Villeurbanne, Auvergne-Rhône-Alpes, France

This paper presents the full haploid genome of the ichneumonid parasitoid wasp Ischnus inquisitor. In the introduction, the authors provide an appropriate background to the biology of the wasp, including information on its host, pre-imaginal development, and geographic distribution. They took advantage of the haplo-diploid genetic system to sequence a haploid male using both long-read and Hi-C Illumina reads, enabling them to produce a highly contiguous assembly organised into chromosomes. The protocol used is described in detail, and the data are publicly available.

 Minor comments:

 It would have been useful to include a few words on the extra-contact that can be observed in the contact map (not only involving car 13, but all the others too). Do these regions contain repeated sequences? 

 In Fig. 4, there is no apparent green curve.

Are sufficient details of methods and materials provided to allow replication by others?

Yes

Is the rationale for creating the dataset(s) clearly described?

Yes

Are the datasets clearly presented in a useable and accessible format?

Yes

Are the protocols appropriate and is the work technically sound?

Yes

Reviewer Expertise:

insect genomics

We confirm that we have read this submission and believe that we have an appropriate level of expertise to confirm that it is of an acceptable scientific standard.

# Reviewer report: Reviewer response for version 1

**DOI:** 10.21956/wellcomeopenres.27254.r139043

**Published:** 2025-12-03

**Authors:** Marta Coronado-Zamora, Vicente Chillida

**Affiliations:** 1 Autonomous University of Barcelona Department of Genetics and Microbiology (Ringgold ID: 196650), Bellaterra, Catalonia, Spain; 2 Institute of Evolutionary Biology, Barcelona, Spain

This data note describes a high-quality chromosomal assembly of the ichneunomid wasp *Ischnus inquisitorius*. The genome data are available at NCBI and data is accessible as checked in November 2025.

**Comments:**

- For the DNA extraction, ratios 260/280 and 260/230 were within acceptable ranges. However, for the RNA-seq sample, although the text states the use of Nanodrop, Qubit, and Agilent RNA 6000 Pico kit, neither the RNA concentration nor any RNA integrity metrics are reported.

- The other specimen collected for RNA-sequencing was also a male?
- For what purpose the RNA seq was produced? is not mentioned in the text. 
- What is the sex-determination system in this species? Why could not the sex chromosomes be assembled?
- There are some software cited in Table 5 but not mentioned in the main text: Cooler, BEDTools, fasta_windows, and Oatk

Are sufficient details of methods and materials provided to allow replication by others?

Yes

Is the rationale for creating the dataset(s) clearly described?

Yes

Are the datasets clearly presented in a useable and accessible format?

Yes

Are the protocols appropriate and is the work technically sound?

Yes

Reviewer Expertise:

population genetics, bioinformatics, genomics, transposable elements

We confirm that we have read this submission and believe that we have an appropriate level of expertise to confirm that it is of an acceptable scientific standard.
